# Extracellular vesicles in β cell biology: Role of lipids in vesicle biogenesis, cargo, and intercellular signaling

**DOI:** 10.1016/j.molmet.2022.101545

**Published:** 2022-07-08

**Authors:** Rebecca S. Aguirre, Abhishek Kulkarni, Matthew W. Becker, Xiaoyong Lei, Soumyadeep Sarkar, Sasanka Ramanadham, Edward A. Phelps, Ernesto S. Nakayasu, Emily K. Sims, Raghavendra G. Mirmira

**Affiliations:** 1 Department of Pediatrics, Baylor College of Medicine, Houston, TX, USA; 2 Department of Medicine and the Kovler Diabetes Center, The University of Chicago, Chicago, IL, USA; 3 J. Crayton Pruitt Family Department of Biomedical Engineering, University of Florida, Gainesville, FL, USA; 4 Department of Cell, Developmental, and Integrative Biology & The Comprehensive Diabetes Center, University of Alabama at Birmingham, Birmingham, AL, USA; 5 Biological Sciences Division, Pacific Northwest National Laboratory, Richland, WA, USA; 6 Department of Pediatrics and the Center for Diabetes and Metabolic Diseases, Indiana University School of Medicine, Indianapolis, IN, USA

**Keywords:** Islet, Diabetes, Extracellular vesicles, Lipids

## Abstract

**Background:**

Type 1 diabetes (T1D) is a complex autoimmune disorder whose pathogenesis involves an intricate interplay between β cells of the pancreatic islet, other islet cells, and cells of the immune system. Direct intercellular communication within the islet occurs via cell surface proteins and indirect intercellular communication has traditionally been seen as occurring via secreted proteins (e.g., endocrine hormones and cytokines). However, recent literature suggests that extracellular vesicles (EVs) secreted by β cells constitute an additional and biologically important mechanism for transmitting signals to within the islet.

**Scope of review:**

This review summarizes the general mechanisms of EV formation, with a particular focus on how lipids and lipid signaling pathways influence their formation and cargo. We review the implications of EV release from β cells for T1D pathogenesis, how EVs and their cargo might be leveraged as biomarkers of this process, and how EVs might be engineered as a therapeutic candidate to counter T1D outcomes.

**Major conclusions:**

Islet β cells have been viewed as initiators and propagators of the cellular circuit giving rise to autoimmunity in T1D. In this context, emerging literature suggests that EVs may represent a conduit for communication that holds more comprehensive messaging about the β cells from which they arise. As the field of EV biology advances, it opens the possibility that intervening with EV formation and cargo loading could be a novel disease-modifying approach in T1D.

## 1 Introduction

Diabetes is a global epidemic affecting over 300 million people worldwide and is expected to increase by 50% by 2030 [1]. Clinically, diabetes has been classified as either emanating from autoimmunity against the insulin-producing β cell (type 1 diabetes, T1D) or from an inability of the β cell to compensate for peripheral tissue insulin resistance (type 2 diabetes, T2D). It is now clear that molecular and phenotypic diversity exists across these two forms such that this simplistic categorization has necessitated more granular refinement. Nevertheless, a common denominator across virtually all classifications of diabetes is the failure of sufficient insulin secretion from β cells, whether due to reduced β cell mass or β cell dysfunction. At the molecular level, extracellular signals (e.g. viruses, inflammatory cytokines, free fatty acids, hyperglycemia) trigger intracellular insults (e.g. endoplasmic reticulum stress, oxidative stress, mitochondrial decompensation, DNA damage) that ultimately lead to cellular senescence, dysfunction, and/or death [2]. Whereas this cell-autonomous perspective of β cell loss has garnered broad acceptance in the field, an increased focus on the islet microenvironment has led to an appreciation of intercellular communication serving to disseminate messages between neighboring cells [2]. Such communication between cells appears to be a means to propagate and amplify signaling that can regulate cellular mass, survival or function. To date, several modes of intercellular communication between β cells and other local cell types (endocrine cells, cells of the immune system, etc.) have been demonstrated, including the release of soluble factors (e.g. senescence-associated secretory phenotype) [3] and direct cell–cell communication (e.g. PD-L1/PD-1 interactions) [4]. More recently, the potential of extracellular vesicles (EVs) as conduits to deliver nucleic acid and protein cargo to nearby cells has gained momentum. In this review, we discuss the pathways that give rise to EVs, the nature of their cargo, and their potential for communication, as biomarkers of β cell stress, and for engineering them as therapeutic vehicles. Particular attention is paid to the role of lipids in these processes.

## 2 EV subtypes and lipid pathways leading to EV formation

**Figure 1:**
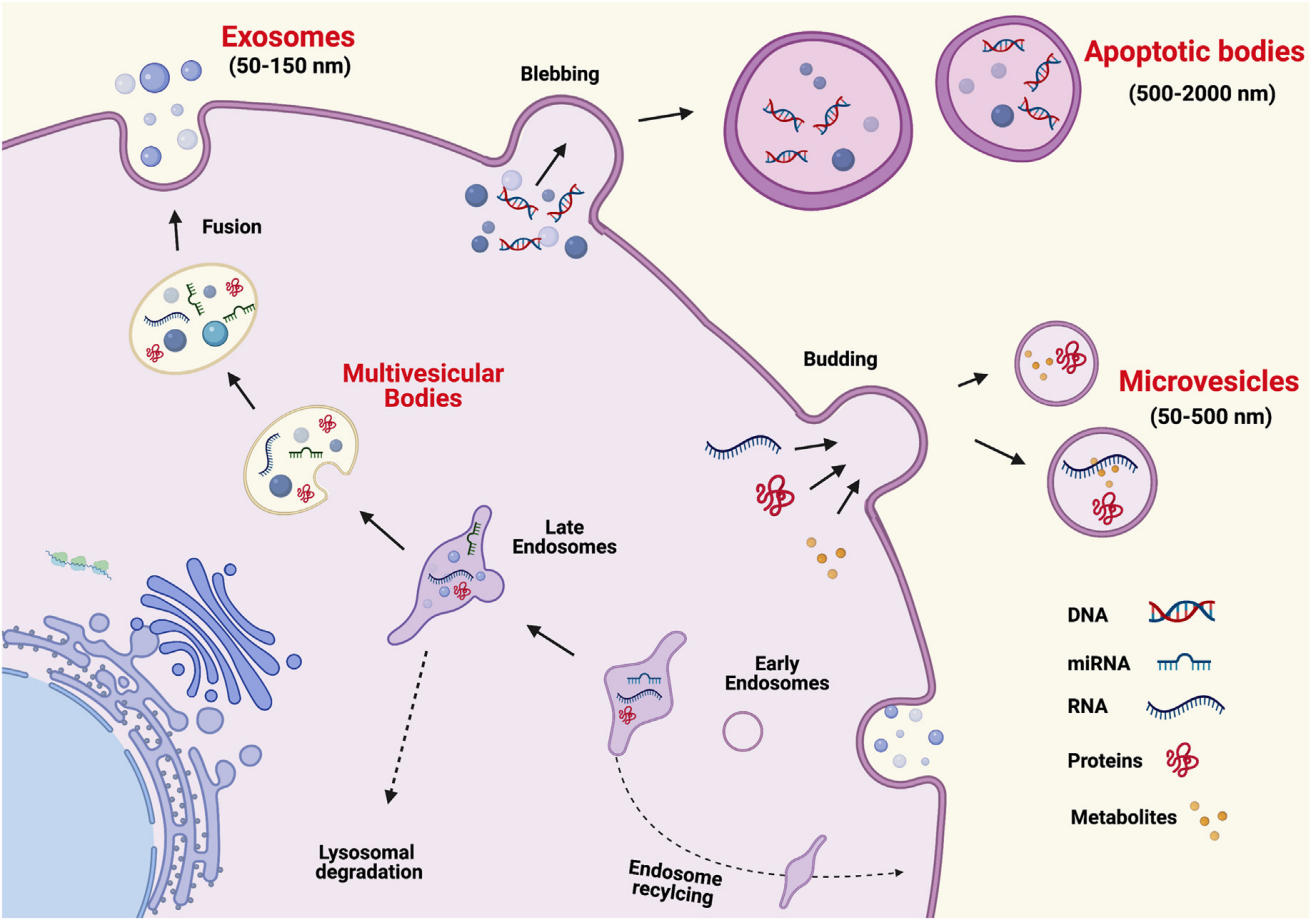
**Biogenesis and secretion of extracellular vesicles (EVs)**. Extracellular vesicles consist of at least three subtypes, exosomes, microvesicles, and apoptotic bodies. The figure shows in the intracellular biogenesis of each subtype and the potential cargo contents of each.

Extracellular vesicles (EVs) are broadly classified into 3 major categories: exosomes, microvesicles, and apoptotic bodies, each of which has distinct intracellular origins (see Figure 1). Exosomes (30–200 nm diameter) and microvesicles (100–1000 nm diameter) are often distinguished based upon size as well as mechanism of formation, which occurs via orchestrated intracellular pathways. Apoptotic bodies (>1000 nm diameter) are derived from the blebbing of cells undergoing apoptosis [5]. Exosomes are generated via an endocytic pathway in which intracellular multivesicular bodies fuse with the plasma membrane and release their intraluminal vesicles. Microvesicles, by contrast, are generated from direct budding of the plasma membrane [6]. The discrete distinction based upon formation may be an over-simplification, as overlapping mechanisms can regulate the formation of both exosomes and microvesicles, with some proposing to categorize only on size (and not mechanism of formation) by labeling the vesicles as “small EVs” (usually <100 nm-200 nm) or “large EVs” (usually >200 nm) [5,7,8].

### 2.1 Formation of exosomes via the endosome pathway

The overall fate of the endosomal pathway can be divided into either reutilization/recycling or degradation [9]. Endosomes are formed by endocytosis, the process whereby extracellular and plasma membrane components are internalized into the cell [9]. These “early endosomes” are then directed to different cellular components such as the trans-golgi network, lysosomes for degradation and recycling, to form late endosomes, or back to the plasma membrane (Figure 1) [10]. The majority of endosomes are recycled back to the plasma membrane but a small number are targeted for autophagy, a degradative process that clears defective proteins and organelles to maintain intracellular homeostasis [11]. The endosomal sorting complex required for transport (ESCRT) protein complexes 0-III are fundamental in the targeting of the endosomes for one pathway or another.

Endosomes that do not quickly recycle back to the plasma membrane or are not targeted to lysosomes for degradation form multivesicular bodies containing intraluminal vesicles [12]. When the multivesicular bodies fuse with the plasma membrane, the released intraluminal vesicles become exosomes [13]. Exosome biogenesis is a dynamic process whereby the contents of the original early endosome may be distinct from the contents released as exosomes. Exosome generation and autophagy pathways overlap with multiple layers of regulation and complexity that are only beginning to be understood [[11], [12], [13]]. Overlapping pathways are speculated to exist, but depending upon cellular conditions, the intraluminal vesicles are shuttled either for release (exosomes) or to lysosomes for autophagy [14]. For example, RAS GTPase superfamily members, specifically Rab GTPases transport and target vesicles intracellularly for endosomal recycling, to lysosomes for degradation, or to secretion as exosomes [9]. Each Rab protein is involved in a specific transport step, with greater than 28 Rab isoforms characterized in exosomes. Most of these are involved in the endocytic pathway, although some are involved in endoplasmic reticulum (ER)-Golgi transport [10]. The three Rab proteins most frequently linked to exosome biogenesis and secretion are: Rab11, Rab35 and Rab27, whose presence direct the multivesicular body to plasma membrane fusion and exosome release. In contrast, Rab7 targets the multivesicular body to lysosomes and subsequent degradation [11]. Table 1 summarizes key proteins that have been described and their functions in extracellular vesicle biogenesis.

**Table 1:** Selection of Key Proteins in Extracellular Vesicle Biogenesis and Lipid Signaling.

Protein Complex or Protein	Role	References
ESCRT	Targets endosomes to a pathway	[11,194]
ESCRT-0 (with Hrs)	Recognizes ubiquitinylated cargo and sorts to PI3P rich vesicles	[9]
ESCRT-1	With ESCRT-2, generates membrane budding inward in endosomes to form intraluminal vesicles, recruits ESCRT-0-ubiquitin domains to buds	[195]
ESCRT-2	With ESCRT-1, generates membrane budding inward in endosomes to form intraluminal vesicles, recruits ESCRT-0-ubiquitin domains to buds	[195]
ESCRT-3	Cleaves membrane buds to form intraluminal vesicles	[195]
Vacuolar protein sorting (Vps)4	Interacts with ESCRT I-III and oxysterol-binding protein; may regulate facilitate miRNAs in exosomes that modulate PI3K/Akt pathway	[22,23,196]
TSG101	ESCRT machinery protein, interacts with ARRDC1, participates in multivesicular body sorting via binding with ALIX, mediates the release of microvesicles containing TSG101, ARRDC1 and other cellular proteins	[15,197]
Arrestin domain-containing protein 1 (ARRDC1)	Interacts with TSG101, mediates the release of microvesicles containing TSG101, ARRDC1, mitochondrial proteins and other cellular proteins; mediates release of exosomes containing proteins implicated in apoptosis	[15,198]
Charged multivesicular body protein (CHMP)	Participates in multivesicular body sorting via binding with ALIX	[197]
Rab GTPases	Transport of endosomes	[9]
Rab11	Targets multivesicular body to plasma membrane for fusion and exosome release	[10,199]
Rab27	Targets multivesicular body to plasma membrane for fusion and exosome release	[10,200]
Rab35	Targets multivesicular body to plasma membrane for fusion and exosome release	[10,201]
Rab22A	Selective recruitment of proteins to microvesicles under hypoxic conditions	[16]
Alg2-interacting protein X (Alix)	Recruited to endosomal membrane via domain that binds BMP; with LBPA, induces formation of endosome membrane invaginations; participates in multivesicular body sorting via binding with TSG101 and CHMP4, interacts with syntenin to support intraluminal vesicle budding	[197,202,203]
Acid ceramidase	Converts ceramide to sphingosine and fatty acids in lysosomes; inhibition increases exosome release	[38,204]
ADP-Ribosylation Factor 6 (ARF6)	GTPase; activates phospholipase D2 & PIP5K; interacts with syntenin; regulates intraluminal vesicle budding and exosome production; regulates selective recruitment of proteins into microvesicles	[14,203,205]
ATP-binding cassette transporter A3	Reduces exosome formation; mediates transport of choline-phospholipids into intracellular vesicles (possible connection to exosome formation?)	[206,207]
Diacyl glycerol (DAG) kinase a	Catalyzes conversion of DAG to phosphatidic acid; inhibits exosome secretion (by reducing DAG levels)	[208]
Flotillins	Modify exosomal cargo	[34,41]
Phosphatidylinositol 4-phosphate 5-kinase (PIP5K)	Catalyzes phosphatidylinositol 4-phosphate (PI4P) phosphorylation to form phosphatidylinositol 4,5-bisphosphonate (PI4,5P_2_), Activated by ARF6 and phosphatidic acid	[209]
Phospholipase D2	Catalyzes hydrolysis of phosphatidylcholine to produce phosphatidic acid; involved in formation of exosomes with syntenin, ALIX, CD63, regulator of intraluminal vesicle budding and exosome production	[205,210,211]
PIKfyve	converts PI3P to either PI(3,5)P2 or PI5P	[20]
Sorting nexin proteins (SNX)	Interact with PI3P and enable transport to trans-golgi or plasma membrane	[20]
Sphingomyelinases (SMase)	Hydrolyze sphingomyelins to produce ceramide	[24]
Syndecans	Ubiquitous transmembrane proteins, enriched in CD63^+^, flotillin-1^-^ exosomes, bind with syntenin, likely recruit syntenin-ALIX and support membrane budding	[203]
Syntenin	Interacts with ALIX, syndecans to support formation of intraluminal vesicles	[203]
Tetraspanins	Participate in exosome biogenesis; interact with other transmembrane receptors, within themselves and with integrins and other proteins; often form tetraspanin-enriched microdomains	[40]
Vps24	Binds with PI(3,5)P2 to target endosomes to EVs	[20]

### 2.2 Pathways leading to release of microvesicles

Localized changes in the plasma membrane that cause direct outward blebbing of the membrane lead to the generation of a microvesicle [12]. Similar to exosomes, microvesicle biogenesis is a regulated process with selected cargo that differs based on the cellular microenvironment [13]. One regulator is the small GTP-binding protein ADP-Ribosylation Factor 6 (ARF6), which initiates a phosphorylation cascade required for microvesicle shedding [14]. Another regulator, TSG101 (tumor susceptibility gene), is an ESCRT machinery protein that interacts with arrestin domain-containing protein 1 (ARRDC1) in order to mediate the release of microvesicles containing TSG101, ARRDC1 and other cellular proteins [15]. Rab GTPases are also involved in microvesicle shedding, especially under hypoxic conditions. Specifically, Rab22A expression increases after the activation of hypoxia-inducible factors and co-localizes with budding microvesicles [16]. TNFα treatment also changes the miRNA cargo of microvesicles released from endothelial cells [17]. While the molecular mechanisms of microvesicle biogenesis are not as well-characterized as exosome biogenesis [18], multiple layers of regulation are clearly involved and a significant degree of overlap in protein families and pathways are likely at play.

### 2.3 Lipids in ESCRT-dependent and ESCRT-independent EV formation pathways

Lipids are an integral component of the ESCRT-dependent system whereby they both recruit appropriate proteins as well as characterize the type of endosome. The phosphatidylinositol phosphates (PIPs) and derivatives recruit cytosolic proteins with key membrane recognition domains, which then participate in transport and possible exosome secretion [19]. The hepatocyte growth factor-regulated tyrosine kinase substrate (Hrs) of the ESCRT-0 complex sorts ubiquitinylated cargo to phosphatidylinositol-3-phosphate (PI3P)-rich endosomal compartments [9]. PI3P interacts with sorting nexin proteins (SNX) to facilitate transfer of the endosome to the trans-Golgi network or plasma membrane [20]. PI3P in turn is converted to either PI(3,5)P2 or PI5P by phosphatidylinositol-3-phosphate 5-kinase (PIKfyve). PI(3,5)P2 is a major component of late endosomes and autophagosomes whereas PI5P is a negative regulator of endosomal maturation and blocks degradation of associated proteins [20,21]. The fate of PI(3,5)P2 containing endosomes seems to be related to whether PI(3,5)P2 binds to vacuolar protein sorting (Vps) 24 (targeting to EVs) or the transient receptor potential cation channel 1 (TRPML-1) (targeting to lysosomes/degradation) [20]. Another Vps (Vps4) associates with ESCRT complexes I-III and (in yeast) has been shown to interact with oxysterol binding proteins, suggesting that oxysterols are implicated in EV formation [22,23]. Alg2-interacting protein X (Alix) is involved in both ESCRT-dependent and –independent pathways. It is recruited to the endosomal membrane via a domain that binds the endosomal lipid bis(monoacylglycero)phosphate (BMP), an endosomal lipid [22]. Lipids relevant in EV biogenesis are summarized in Table 2.

**Table 2:** Lipids Subtypes in Extracellular Vesicle Biogenesis.

Stage of Biogenesis	Lipid	Main Function(s)	References
Formation	Bis(monoacylglycero)phosphate (BMP) [a.k.a lysobisphosphatidic acid (LBPA)]	Induces formation of multivesicular bodies, interacts with ALIX, enriched in late endosomes	[22,202,212]
	Ceramides	Promotes formation of multivesicular bodies	[31]
	Cholesterol	Allows for membrane conditions permitting budding	[205]
	Diacylglycerol	Promotes formation of secretory vesicles in the trans-Golgi network; recruits soluble cell membrane proteins and interacts with cytoskeleton	[208]
	Phosphatidic Acid	Regulates membrane curvature and fission; interacts with syntenin and other proteins in intraluminal vesicle budding	[43,211,213,214]
	Phosphatidylinositol 3-phosphate (PI3P)	Feature of early endosomes and multivesicular bodies; interacts with Rabs, SNX's, HRS, PILfyve	[9,215,216]
Transport and Cargo Sorting	Ceramides	May enrich for certain miRNA cargo	[33]
	Cholesterol	Increases release of flotillin^+^ exosomes (oligodendroglial cells); controls movement of endosomes along microtubules	[41,217]
	Ether lipids	Changes protein composition of EVs	[218]
	Lysophosphatidylcholine	With palmitate increases proinflammatory EV cargo	[219]
	Palmitate	With lysophosphatidylcholine increases proinflammatory EV cargo	[219]
	Phosphatidylserine	Regulates retrograde transport	[215]
	PI3P	Interacts with SNX, facilitates endosomal transfer to the trans-Golgi network or plasma membrane	[20]
	Sphingosine 1-phosphate	Activates inhibitory G protein-coupled S1P receptors on multivesicular bodies (necessary to target CD63, CD81, flotillin to intraluminal vesicles destined for plasma membrane)	[220]
	Cholesterol	Increases exosome release, Induces fusion of multivesicular vesicles with cell membrane	[147,221]
	Ether Lipids	Increases exosome release	[218]
	Phosphatidylinositol-3,5-bisphosphonate [PI(3,5)P_2_]	Component of late endosomes; interacts with Vps24 to target vesicles to EVs; high levels may target vesicles to autophagy/lysosomes	[20,24,215,222]

Prominent among the ESCRT-independent pathways is the ceramide-dependent pathway. Ceramides (Cer) are sphingolipids that are generated in the ER by hydrolysis of sphingomyelin by sphingomyelinases (SMases) or lysosomes [24]. Ceramides are the central precursors for a family of sphingolipids that includes sphingomyelins and sphingosine phosphate (S1P). Ceramides can be generated via multiple pathways: *de novo* [25,26], sphingomyelin hydrolysis by acid (A) or neutral (N) SMases [27], or salvage [28]. The major ceramides in exosomes are the C18:0-Cer and C24:1-Cer molecular species [29,30] and they are proposed to enable membrane curvature to facilitate inward budding of the vesicles.

In a mouse oligodendroglial cell line, exosome formation is decreased both by knockdown or chemical inhibition of NSMase [31]. The importance of ceramide in EV formation (via NSMase) has been confirmed in several other cell lines, although it does not appear to be a uniform trait, since reduced SMase does not seem to affect exosome numbers in prostate nor melanoma cells [[32], [33], [34], [35]]. In polarized epithelial cells, ceramides are involved in differential exosome secretion in which basolateral but not apical exosome release depends upon ceramides [36]. Notably, while neutral SMase plays a role in exosome biogenesis, acidic SMase triggers microvesicle release in glial cells [37].

### 2.4 Lipid rafts

Lipid rafts, or lipid microdomains, are membrane sections enriched in sterols, sphingolipids and glycosylphosphatidylinositol (GPI)-anchored proteins. They generally function in forming membrane invaginations, either via structural changes induced by lipid raft components and/or by protein recruitment and complex formation [38,39]. Among these recruited proteins are the tetraspanins, (also known as transmembrane 4 superfamily (TM4SF) proteins) and flotillins. Tetraspanins, which are intimately associated with exosomes, are thought to participate in exosome biogenesis, although the exact mechanisms have yet to be elucidated. These proteins interact with other proteins, including other tetraspanins, transmembrane receptors, and integrins, often forming distinct membrane regions called tetraspanin-enriched microdomains [40]. Flotillins do not seem to be directly involved in exosome release, but rather in modifying exosomal cargo [34,41].

Within lipid rafts exist planar lipid rafts, with similar architecture as surrounding membranes, and invaginated (caveolae) lipid rafts, which have a distinct appearance from the rest of the membrane [39]. Caveolae lipid rafts are non-clathrin-coated pits rich in the membrane protein caveolin and participate in intracellular signaling, vesicle trafficking, and cell migration. The caveolin protein Cav1 has been described in exosomes from several cancer cell lines and in exosomes harvested from serum of melanoma patients [42]. Given the location of caveolin in lipid rafts and role in cellular signaling and endocytosis, it likely contributes to intraluminal vesicle sorting and exosome formation [39].

### 2.5 Other relevant lipids in EV biogenesis

In addition to the aforementioned lipids, multiple other lipids are known or suspected to participate in different stages of EV biogenesis. Table 1 outlines many of the proteins involved in the formation of these lipids and Table 2 details individual lipids contributing to EV biogenesis. Importantly, the role of a particular lipid may vary depending upon cell type or physiological state (i.e. healthy, stressed, diseased). Another complicating factor is that many studies use exosome number as the measured variable. Thus, while one can conclude that a compound inhibits or promotes total EV release, which stage of EV biogenesis and release is involved still needs to be elucidated.

### 2.6 Lipid composition of EVs

Lipid content (relative to proteins) in exosomes is enriched 8.4-fold as compared the cell from which they are derived [43]. The lipid composition of EVs depends both upon cell of origin and health or disease status (i.e. EVs from malignant cells may have different compositions than nonmalignant cells) and likely also differs between primary cells and immortal cell lines [19,44]. Mass spectrometry quantification of the lipidomes of a prostate cell line (PC-3) and its released exosomes reveals that exosomes are enriched in glycosphingolipids, sphingomyelin, cholesterol and phosphatidylserine [43]. Collation of exosome lipid composition from multiple studies shows that phosphatidylethanolamine content is similar in cells and exosomes whereas phosphatidylcholine and phosphatidylinositol contents are lower in exosomes compared to cells [19], perhaps to facilitate target cell uptake [45] or due to the role of these lipids in EV biogenesis (Table 2). In 3T3-L1 adipocytes, phospholipids, sphingolipids, and glycerolipids are similar between the smaller EVs (mostly <100 nm) and large EVs (100–200 nm). However, cholesterol is more enriched in smaller EVs and annexin V binding (marker of externalized phosphatidylserine) is more enriched in large EVs [46].

In contrast to studies of lipid content in cell line-derived EVs, less information exists from EVs isolated from biofluids. One study examined the EVs obtained from the ejaculate of vasectomized men with EVs separated by differential centrifugation and size-exclusion chromatography into approximately 50 nm and 100 nm populations [47]. In contrast to studies in adipocyte cell lines, cholesterol content was shown not to vary significantly between smaller and larger EV populations; however, hexosylceramide is enriched in the smaller EVs and sphingomyelin species are enriched in larger EVs [47]. Exosomes harvested from urine have been shown to have significant cholesterol content and the lipidome characterized by mass spectrometry can be used to distinguish healthy controls from those with prostate or renal cell carcinoma [48,49]. Lipid content of EVs isolated from human serum was determined by an ultracentrifugation protocol in which large debris and apoptotic bodies were first removed, followed by ultracentrifugation of the supernatant. The lipid content was examined in a total of 7 fractions and varied depending upon the fraction, with the most buoyant fraction being lipid rich but lacking typical EV markers. The EV pellet was enriched with ceramides [50]. To our knowledge, the lipidome of β cell-derived EVs has not been characterized; however, it is possible that there may be a unique lipid signature of these EVs and/or a characteristic signature of stressed β cells.

## 3 Lipid cargo in EVs

**Figure 2:**
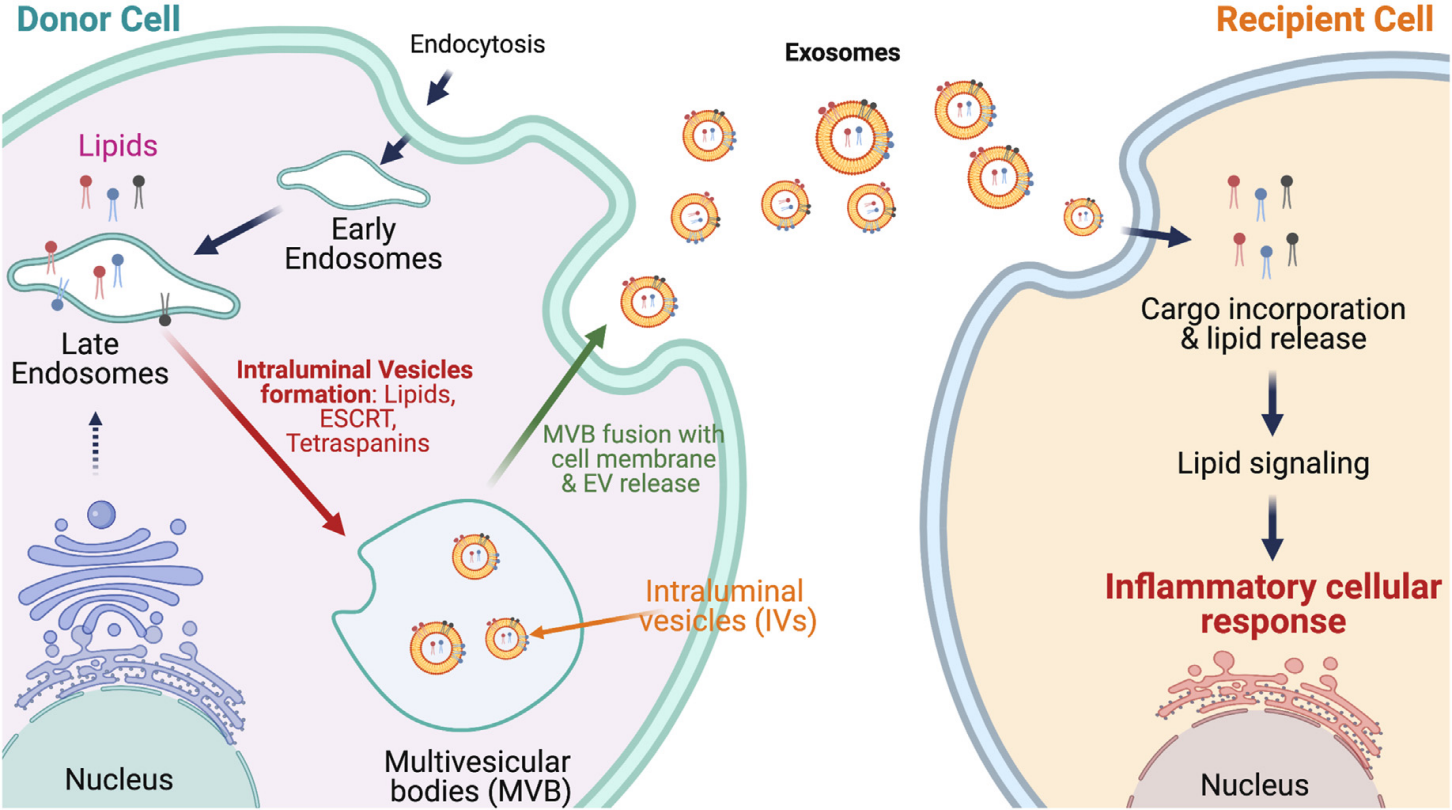
**Lipid incorporation into exosomes and their release to recipient cells.** The figure illustrates incorporation of lipids into exosomes of “donor” cells (*left*), which release these exosomes that then communicate with the neighboring or distant “recipient” cells (*right*).

EVs, initially described as conduits for disposal of undesired cellular material, are now considered to be part of the secretome, serving as carriers of important cargo that can be delivered from a donor cell to a same or different recipient cell type (see Figure 2). Among the cargo includes proteins, enzymes, transcription factors, RNA, miRNA, DNA, and lipids [22,[51], [52], [53], [54], [55]]. Studies during the past decade have revealed that EVs are released from a variety of cells and their cargo participates in a variety of disorders, predominantly cancer, neurological, and metabolic disorders [56,57].

Recent attention to EVs in T1D has been directed at understanding the signaling transmitted by their protein or miRNA cargo. However, whereas the nature of EV-derived lipid signaling has been addressed in other disorders, it has been largely absent in the T1D literature. This section of the review will discuss the important roles of EV lipid cargo in propagating deleterious outcomes and extrapolate their importance in β-to-immune cell communication, which ultimately leads to T1D development.

### 3.1 Ceramides

A distinguishing feature of exosomal membranes, compared with plasma and microvesicle membranes, is the lipid composition, which, as noted previously, is enriched compared to their cell of origin. One of these, the ceramides, represents a class of sphingolipids that has generated much interest [58]. As signaling molecules, ceramides are implicated in inducing apoptosis in multiple systems [59,60]. As one example, treating oligodendroglioma cells with the Th1 pro-inflammatory cytokines TNFα and IFNγ can cause cell death via the formation, and exosomal release, of multiple ceramide molecular species [61,62]. Such shedding of ceramides was proposed to contribute to autoimmune responses that lead to neuronal demyelination. Consistently, TNFα and IFNγ are reported to be elevated in the brains of patients with multiple sclerosis (MS) [63], a disease associated with demyelination, and this is associated with increased accumulation of ceramide molecular species in the cerebrospinal fluid [64].

The neurodegenerative Alzheimer's disease is associated with the accumulation of amyloid-β peptides in the brain. During the progression of this disease, ceramide levels in the human brain increase [[65], [66], [67]]. Silencing NSMase2 using genetic approaches leads to reduced exosomes in the brain, reduced ceramide levels, and decreased plaque burden [68,69], suggesting that ceramide-rich exosomes contribute to Alzheimer's disease pathology. Similarly, Parkinson's disease is associated with aggregation of high molecular weight α-synuclein [70]. Reducing NSMase2 activity leads to decreased transfer of α-synculein aggregates between neuronal-like cells and less accumulation and aggregation of the high molecular weight α-synuclein [71]. While there are several reports of NSMase involvement in a variety of disorders [72], exosomes in the CSF of multiple sclerosis patients were found to contain high SMase activity, which correlated with exosome number and severity of the disease [73].

A recent comprehensive review [74] elegantly discusses the critical role of ceramides and S1P in the function and secretion of exosomes in a cancer milieu. For example, ceramides generated by the NSMase pathway in exosomes from a human multiple myeloma cell line increases exosomal secretion, decreases cell proliferation, and increases caspase-3/9/PARP-mediated cell death [75]. This finding is associated with increases in exosomal abundance of tumor-suppressive miRNAs. All of these outcomes are reversed with GW4869, an inhibitor of NSMase2. Consistently, lower expression of NSMase1 in exosomes has been reported in patients with hepatocellular carcinoma and is associated with poor long-term survival of those patients [76]. Utilizing hepatocellular carcinoma cell lines, the authors demonstrate that exosomal NSMase1 reduces hepatocellular carcinoma growth by decreasing the ratio of sphingomyelins to ceramides. Inflammation of the gut, promoted by bacterial infection, can lead to colon cancer. The enteropathogenic bacteria are proposed to stimulate intestinal epithelium to produce exosomes that are enriched in S1P and prostaglandin E_2_ (PGE_2_) lipids [77]. Through induction of inflammatory Th17 cells, S1P is thought to promote tumor cell growth, whereas PGE2 participates in recruitment and proliferation of Th17 cells.

Exosomal ceramides also are reported to play a role in mitigating atopic dermatitis, a systemic inflammatory disease that is associated with epidermal barrier disruption [78]. In this study, administration of exosomes from human adipose tissue-derived mesenchymal stem cells to mice treated with oxazolone to induce dermatitis was found to reduce inflammatory cytokine levels and restore epidermal barrier functions. These outcomes correlate with decreased ceramide production via the *de novo* pathway and increasing anti-apoptotic S1P via the salvage pathway. In addition to ceramides and S1P, other toxic sphingolipids like sulfatides and psychosine are under consideration as being shuttled from sick to healthy cells by exosomes, thereby spreading the disease [79].

### 3.2 Eicosanoids

Another class of lipids that are synthesized within and shuttled by EVs are eicosanoids, bioactive oxygenated lipid metabolites of arachidonic acid [54,80]. Arachidonic acid is hydrolyzed from membrane phospholipids by phospholipases A_2_ (PLA_2_) [81,82]. The most abundant eicosanoids in EVs are prostaglandins and leukotrienes, which are also among the most inflammatory lipids [81]. Early studies identified the involvement of secreted PLA_2_, PGE_2_, and lysophosphatidylcholine [83,84], the other product generated by PLA_2_ action, in maturation of dendritic cells. Subsequent studies revealed the presence of lysophosphatidylcholine and phospholipase D in exosomes from a basophilic leukemia cell line (RBL-2H3) [85,86]. Further analyses [87] showed that the cargo load of these exosomes includes prostaglandins, such as PGE_2_ and 15d-PGJ_2_, a PPARγ agonist. Moreover, the concentrations (micromolar) of PGs in the exosomes are sufficient to affect signaling. In another study, PGE_2_ delivered by IDENs (intestinal mucus-derived exosome-like nanoparticles) to the liver was shown to induce anergy in natural killer T cells and reduce their ability to respond to foreign antigens [88]. In addition to its ability to affect communication between cells as EV cargo, PGE_2_ through EP_4_ receptor signaling can modulate EV sorting [89]. In this regard, clinical chemoresistant breast cancer carcinoma cells express high COX2-PGE_2_-EP_4_ signaling [90] and blocking the PGE_2_/EP_4_ signaling in cancer stem cells has been reported to promote conversion of of these cells to non-cancer stem cells [89]. This effect is a consequence of increased EV release of mesenchymal markers and drug transporters from cancer stem cells, reducing the number of chemoresistant cells that continue to proliferate. Paradoxically, the COX2 inhibitor celecoxib, which reduces COX2 mRNA in several cancer cells, increases COX2 accumulation in exosomes of lung cancer cells [91]. Recipient cells of these exosomes produced higher PGE_2_ and vascular endothelial growth factor, thus spreading inflammatory responses.

Metabolism of arachidonic acid by 5-lipoxygenase leads to generation of leukotrienes. Among them, LTB4 has garnered much attention owing to its varied roles, including in recruitment of neutrophils and other leukocytes in autoimmune diseases [92,93]. Exosomes contain the enzyme machinery to synthesize LTB4 [94] and inhibition of the enzymes decreases exosomal LTB4 and results in loss of directionality during neutrophil migration [95]. Given the short half-life (1 min) of LTB4 *in vivo*, stability and longevity of LTB4 is achieved through packaging in EVs [96] and its time-dependent release from exosomes serves as a signal to recruit neutrophils [97].

Metabolism of arachidonic acid by 12-lipoxygenase (12-LOX) leads to generation of 12S-HETE [98,99], which promotes invasion and metastasis of tumors [[100], [101], [102]]. Platelets produce 12-LOX [103] and can deliver 12-LOX packaged in EVs to colon cancer cells, leading to an increased production of 12S-HETE and its esterification into cell membrane phospholipids [104]. A further example of a role for platelet EV cargo 12-LOX was demonstrated in autoimmune arthritis [105]. During inflammation, secreted PLA2-IIA is expressed in inflammatory exudates [106] and catalyzes hydrolysis of arachidonic acid, which is then metabolized to 12S-HETE by 12-LOX in the platelet EVs. When neutrophils reach the inflammatory site, signaling by 12S-HETE via BLT2 receptors on activated neutrophils triggers internalization of platelet EV cargo, which enhances inflammation [105].

## 4 Other signaling cargo in EVs

In recognizing the potential importance of cell-to-cell communication in T1D development, much of the work characterizing β cell EVs has focused on mRNAs, miRNAs, and protein cargo. Independent studies have identified a variety of these species in β cell EVs that can impact β cell function and survival including: miR-127 which can inhibit β cell proliferation and insulin secretion [107]; mRNAs (VEGF-A, eNOS) and miRNAs (27b, 126, 130, 296) that contribute to β cell function and islet endothelial cell angiogenesis, a key for engraftment of transplanted islets [108]; miR-21–5p, which is induced during inflammation and can increase β cell apoptosis in T1D [109,110]; 19 miRNAs and 133 mRNAs [111] and cytokines [112] that are differentially expressed by islets under proinflammatory conditions; and accumulation of the spliced form of XBP1 mRNA, which correlates with ER stress and apoptosis [113]. It has also been suggested that islet graft rejection may be a consequence of major histocompatibility complex (MHC) molecules carried by donor islet EVs that are recognized by recipient antigen presenting cells (APCs) and immune cells [114].

In other studies, EVs from various preparations have been related to consequences of islet stress in diabetes, although the precise EV cargo was not identified. For example, human bone marrow-derived mesenchymal stem cell EVs can induce tolerogenic dendritic cells to reduce inflammation and mitigate T1D progression [115]; pancreatic cancer cell-derived EVs induce ER stress genes and β cell dysfunction [116]; human islet EVs containing mRNA of differentiation factors NGN3, MAFA, and PDX1 upregulate C-peptide levels in iPSC clusters [117], suppress human islet amyloid polypeptide amyloid formation that occurs in the islets of patients with T2D [118], and trigger immune responses and activate PBMCs [119]; human pancreas-derived mesenchymal stem cells facilitate improved glucose tolerance, increases in β cell number and insulin secretion in streptozotocin-treated mice [120]; human umbilical cord mesenchymal stem cells protect β cells from hypoxia-induced apoptosis [121]; healthy and human lean adipocyte-derived EVs but not inflamed or obese adipose explant-derived EVs improve β cell function and survival [122]; and EVs from cytokine-exposed β cells promote the interaction of EV-derived CXCL10 with recipient β cell CXCR3 receptor to induce a proinflammatory islet transcriptome and β cell dysfunction [123].

## 5 β-cell stress, EV formation, and connection to T1D pathogenesis

Little is known regarding the intersection of β cell stress and pathways that lead to EV formation, although common players exist in both diabetes pathogenesis and EV biogenesis. As one example, cytokines such as IL-1β or TNFα have been shown to activate the sphingomyelin/ceramide pathway or increase ceramide content [124]. Proinflammatory cytokines also activate or upregulate SMases, leading to increased ceramide β cell content with associated ER stress, mitochondrial damage and caspase-3 activation [124,125]. It is conceivable that these same pathways influencing ceramide content in β cells could also affect EV biogenesis and EV properties. Ceramide-rich EVs induce apoptosis in astrocytes [30] as well as in IFNγ-primed oligodendrocytes [61], suggesting that similar mechanisms could be at play in other diseases, including diabetes. Another possible impact of stress-induced alterations in β cell lipid pathways involves dysregulation of intracellular endosome trafficking. Defects in autophagy have been associated with the development of both T1D and T2D [126]. Since significant overlap exists in the pathways leading to exosome formation and targeting of endosomes to degradation, reduced autophagy in T1D might be associated with changes in multivesicular endosome trafficking and exosome formation. Similarly, the induction of stress granule production by ER stress and the integrated stress response is thought to alter the potential content of EVs as they are shuttled through the endosome pathway. Stress granules are non-membranous cellular inclusions that contain translationally inhibited mRNAs and RNA binding proteins [127], and it has been shown that hnRNPs present in stress granules can be identified in EVs [128].

**Figure 3:**
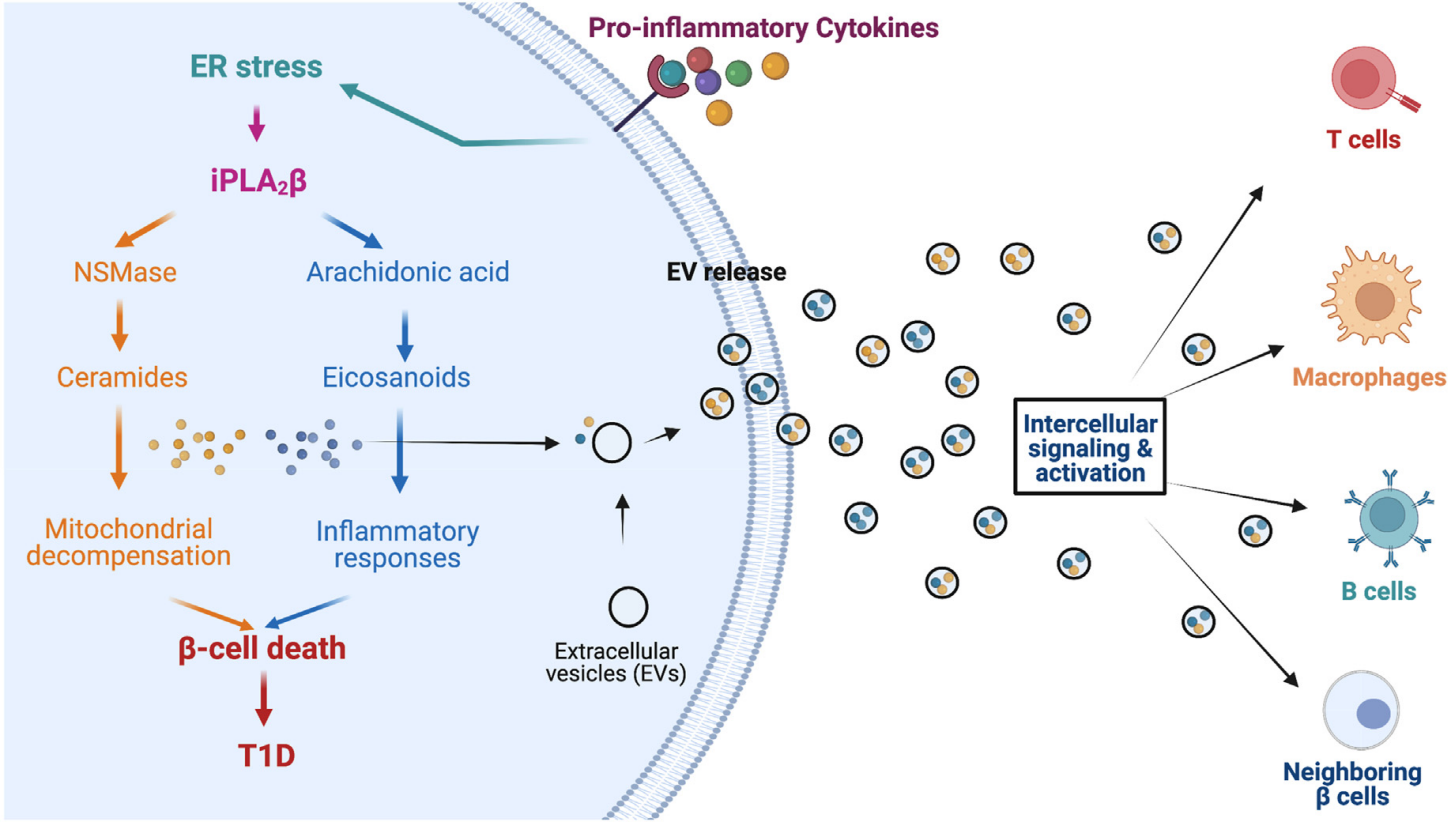
**Activation of iPLA_2_β signaling and incorporation of lipid products into exosomes.** The figure depicts that stressed β cells in the setting of type 1 diabetes induce ER stress and iPLA_2_β activity, whose downstream products lead to both β cell death and are incorporated into exosomes for intercellular signaling to neighboring islet cells and immune cells.

ER stress-induced β cell death is associated with increases in ceramide generation via hydrolysis of sphingomyelins by NSMase. Accumulations in ceramides cause decompensation of the mitochondria, leading to release of cytochrome C into the cytosol, inducing caspase-3-mediated β cell apoptosis [[129], [130], [131], [132]]. More recently, it was reported that the development of T1D is associated with increased production of proinflammatory eicosanoids by immune cells [133,134]. These outcomes are likely mediated by activation of the Ca^2+^-independent phospholipase A_2_β (iPLA_2_β). Given the observations that ER stress in β cells precedes T1D onset [135,136] and the literature available to date on EV lipid cargo, we posit that cells integral to inducing β cell death in T1D communicate with each other, in part through lipid signals delivered by EVs, as illustrated in Figure 3.

The impact of the lipid load in β cell-derived EVs has received little attention, as evidence by a recent report in which a survey of EV lipid content across various donor cells did not include the β cell [137]. Notably, a study performed with RBL-2H3 mast cells revealed that, in addition to prostaglandins, the EVs from these cells contain a truncated iPLA_2_β protein [87]. It has also been previously reported that this isoform is generated through caspase 3-mediated cleavage of iPLA_2_β and that the truncated iPLA_2_β protein manifests greater specific activity [133,134]. Collectively, these observations provide rationale to examine the contribution of EV-derived lipid signaling shuttled between donor/recipient β cells and immune cells. As can be deduced from the reports of EV-lipid signaling in various contexts above, such investigations will undoubtedly provide insights to new therapeutic avenues and identify lipid-related biomarkers of T1D onset.

## 6 Extracellular vesicle components as potential T1D biomarkers

For clinical trial purposes, a staging scheme for T1D has been recently described [138] and includes pre-symptomatic periods of auto-antibody positivity but without overt hyperglycemia. Therefore, the development of biomarkers is deemed crucial for disease prognosis and for the identification of patients who would benefit from preventative therapies. EVs have potential as biomarkers, as they carry cellular components from the cells from which they are derived that serve as molecular “signatures” of the disease process. Because specific molecular species (miRNAs, lipids, metabolites) are enriched in EVs, interrogation of EVs for these molecules might lead to the identification of biomarkers that would be otherwise diluted and lost to detection in circulating plasma. By example, in prostate cancer, urinary EVs have been shown to carry RNAs that serve as signatures of disease outcomes [139]. Similarly, in T1D, diverse components of β cell-derived EVs, including miRNA, DNA, protein, and metabolites have been identified [140,141]. It is reported that rat and human pancreatic β cells under inflammatory stress release EVs containing GAD65, IA2, and proinsulin that can subsequently can activate dendritic cells and propagate adaptive immunity [142]. Studies have shown β cell EV production and cargo are altered upon proinflammatory cytokine exposure [111,143], reinforcing their potential to harbor biomarkers. Further, various clinical studies report differential expression of various miRNA in subjects with long-standing T1D compared to healthy controls, among which miR-21–5p is being considered as a biomarker [110,[144], [145], [146]]. With respect to diabetes, one group used lipidomic profiling of urinary exosomes to distinguish individuals with diabetic nephropathy (as compared to controls without nephropathy) and were able to identify 5 lipids as significantly different [147]. Further research is warranted to determine if a single biomarker or a group of biomarkers in these EVs reflect β cell health.

Dysregulation in lipid metabolism has been suggested to precede the onset of autoimmunity in T1D [148]. In this study, serum metabolomics was performed from samples obtained from youth who later progressed to T1D. As compared to healthy controls, youth who later progressed to T1D demonstrated increased levels of proinflammatory lysophosphotidylcholines several months before seroconversion to auto-antibody positivity. Another study demonstrated the utility of sphingolipids as biomarkers in T1D [149]. In that study, using animal models of diabetes, it was demonstrated that sphingosine-1-phosphate is elevated compared to the controls. In addition, diabetic animals demonstrated reductions in plasma levels of cytoprotective omega-9 24:1 (nervonic acid)-containing ceramide, sphingomyelin, and cerebrosides. A metabolomic study from cord blood also demonstrated that levels of phospholipids, mainly phosphatidylcholines and phosphatidylethanolamines, were decreased in T1D subjects long before diagnosis [150]. Finally, a recent study also demonstrated that the iPLA_2_-derived lipids are enhance in T1D subjects compared to healthy controls [151]. These different studies reveal the relevance of lipids as biomarkers of T1D progression. It is unknown if these lipids are also released as EV cargo; however, considering the aforementioned studies in which lipid EV cargo appears to contribute to disease pathogenesis, it is likely that lipid EV cargo contributes to the development of diabetes and could serve as a biomarker.

Whereas EV properties such as their relative stability and protection from circulating protease/nuclease activity are attractive for their use in biomarker development, a major challenge to their clinical utility lies with their isolation. Several biophysical approaches have been employed for the isolation of EVs including ultracentrifugation, density gradient ultracentrifugation, polymer-based precipitation, size-exclusion chromatography, and immunocapture. These techniques all have their advantages, but they also suffer from unique profiles of contaminants. For instance, while ultracentrifugation is technically simple, it results in poor recovery and large compositional variability. Polymer-based precipitation has the advantage that it is a rapid isolation technique allowing for isolation and characterization of large numbers of samples; however, polymers can alter the composition of the EVs, and the levels of polymers required to remove contaminating lipoproteins result in precipitation of a majority of the EVs of interest. Similarly, while size exclusion chromatography is gentle and has excellent recovery and repeatability/reproducibility, EVs share similar hydrodynamic diameters with large plasma lipoproteins, which results in co-fractionation of these distinct populations that can confound compositional analysis [[152], [153], [154], [155]]. This issue remains an ongoing concern that receives continually refined recommendations by the International Society for Extracellular Vesicles [8].

## 7 Bioengineering EVs

With the increasing body of knowledge on factors effecting EV biogenesis, secretion, contents, and (patho)-physiological roles, more research is now being focused on how EVs might be used as therapeutic tools. Indeed, EV-based therapeutics can impart many of the same effects as their cell-based counterparts while avoiding several key drawbacks associated with patient risk, scalability, and cost [156]. The distinct biological structure and function of EVs means that they possess high physiochemical stability [157], innate biocompatibility, and the ability to interact with cells through signaling, fusion, and delivery [158]. Despite these advantages, EVs must still be altered in many cases to complement or enhance their therapeutic applicability. Here, we will discuss several approaches for EV engineering and suggest how they might be applied to T1D, as well as some considerations for translational potential and commercial scalability. We will also discuss how knowledge of lipid EV cargo is crucial for therapeutic applicability.

Of the different approaches for EV functionalization, indirect engineering through changing the parent cells is perhaps the most readily adoptable method, as cell functionalization is a highly established and widely employed field. For example, it was reported that exosomes from human bone marrow mesenchymal stem cells transfected with pshFas-anti-miR-375 silence Fas and miR-375 in human islets to improve viability and function against inflammatory cytokines after transplantation [159]. Although this approach involved gene silencing, a more commonly employed method is to use tetraspanins such as CD9, C63, and CD81, which are enriched in EVs [160], as backbones for fusion proteins intended for EV loading. Tetraspanin fusion proteins are used for a variety of purposes, from investigating secretion [161] and trafficking [162] of EVs, to enabling more flexible engineering of EV surfaces [163] and efficient EV loading of therapeutic cargo [164,165]. Other groups have used Rab5a [166] or lactadherin [167], both also enriched in EVs, as backbones for fusion proteins for EV loading. Recently, it was reported that exosomes derived from HEK293 cells engineered to express an MHC-I/CD81/IL-2 fusion and a CD80/lactadherin fusion were able to selectively deliver co-stimulation and IL-2 to antigen-specific CD8+ T cells [168], highlighting the distinctive role that engineered EVs can play in antigen specific immune interactions. Engineering EVs for T1D therapeutics through cell functionalization therefore seems highly feasible. For example, β cells genetically engineered to express tetraspanin fusions containing immunomodulatory proteins or cytokines could be loaded into EVs that inherently display β cell autoantigens in native MHC. Such EVs could then function as a cell-free, antigen specific immunotherapy against diabetogenic T cells. However, cell-based EV functionalization typically results in low packaging efficiency of therapeutic cargo, which is highlighted by the large knowledge gap in the importance of post-translational modifications in conferring specific properties to proteins and sorting them into EVs [169]. There have been scattered reports of lipid post-translational modifications, such as palmitoylation, that targets a protein to EVs [170]; however, their relevance for EVs in T1D therapeutics remains to be seen.

To overcome packaging inefficiencies, many groups have focused on directly engineering EVs after purification. Direct EV engineering can be accomplished in both passive and active manners to modify the surface with membrane binding species or to encapsulate materials into the vesicle interior. Loading EV interiors with cargo is achieved through membrane permeabilization. Permeabilizing via electroporation is commonly used to load EVs with RNA cargos that then are taken up by target cells to impart therapeutic effects [[171], [172], [173]]. Active EV surface modification often includes some sort of chemical process to alter the lipids or proteins on the surface of EVs, such as through click chemistry [174,175]. Multiple groups have also devised ways to directly load peptide antigen into exosomal MHC [176,177], again highlighting the potential for EVs as antigen specific therapies. Passive surface modification relies on noncovalent interactions including hydrophobic insertions, regularly used to label EVs with hydrophobic dyes for investigating trafficking [[178], [179], [180]], and receptor-ligand binding. In one recent example, an anchor peptide that enables EV targeting of cargo through binding to CD63 was identified [181]. Coupling several of these direct engineering strategies enables a “plug-and-play” approach for internal cargo loading and external decorating to engineer “designer” EVs. For instance, EVs derived from dendritic cells (DCs) could be loaded with therapeutic RNAs like miR-375 [159] and targeted to islets through surface functionalization, or directly loaded with β cell autoantigens and functionalized with immunomodulatory proteins. Indeed, there is already precedence for using DC-derived EVs as therapies for cancer in clinical trials [182].

In addition to creating targeted EV cargo, knowledge of cell membrane lipid components could be used to target therapeutic EVs to specific cell types. The enrichment of phosphatidyl serine in EV membranes has also been exploited experimentally for EV targeting by fusing a phosphatidyl serine-targeting protein to a ligand for cell receptors of interest, thereby targeting the EVs to the desired cell type [183]. The extent to which the lipid composition of the EV membrane participates in specific cellular targeting is not known. However, fusion of EVs with liposomes have shown that different lipids can contribute to increased uptake. The caveat of these studies is that they were only performed *in vitro*, and not *in vivo* [184]. To add to the complexity, a recent study showed that cellular uptake does not automatically indicate integration into the cytosol; the exosomes can be re-released without apparent modification or housed in endosomes for days only to undergo degradation [185].

Any EV-based therapeutic intended for clinical use must pass regulatory rigor and conform to good manufacturing practice (GMP) grade protocols. To this end, several groups have described technologies and protocols for EV manufacturing that adhere to GMP standards [[186], [187], [188]]. Considerations for scalability and quality control are also important. Commercial bioreactors are widely available for large-scale cell expansion, with microcarriers [189] or hollow fiber bioreactors [190] now being used to reduce costs. It is possible to collect EVs from cells cultured in hollow fiber bioreactors [191], demonstrating the field's interest in commercializing EV therapeutics. Furthermore, recombinant EVs as a biological reference tool for quantifying recovery efficiencies of common techniques and normalizing and improving sensitivity of EV counting methods has been described [192]. While there are already several EV-based treatments in clinical trials for various diseases that show great promise [193], technologies such as those described above will be important for widespread, safe adoption of this class of therapeutics.

## 8 Conclusions

EVs represent a new horizon in the study of diabetes pathogenesis. In recent years, it has become apparent that different classes of EVs have different intracellular origins, contain sometimes distinct cargo, and have lipid content that is enriched compared to the cell of origin. Moreover, it is now appreciated that the content of EVs can vary depending on the stress state of the cell. This latter observation has significant ramifications with respect to intercellular communication, thereby allowing neighboring cells to recognize and possibly respond to states of prevailing stress by incorporation of EV cargo. Further elucidation of EV function in health and disease has the potential to leverage EVs as circulating biomarkers of prevailing cellular health and possibly engage engineered EVs for targeted therapeutics. Nevertheless, several challenges remain in leveraging EVs to better understand biology and manipulate pathologic states. These include, but are not limited to, a better understanding of how cargo (lipids, RNA, metabolites) is specifically shuttled into EVs, methods for the reproducible and reliable isolation of EVs, and methods for engineering and cargo loading of EVs for cell-directed therapies. Given the potential promise that EVs hold for therapeutics and diagnostics, recent requests for applications from major granting agencies (e.g. US National Institutes of Health) ensure that EV biology is likely to progress rapidly in the coming years to meet these and other challenges.
